# Safe and efficient transportation of clinical samples for molecular detection of African swine fever virus

**DOI:** 10.3389/fcimb.2025.1630865

**Published:** 2025-09-22

**Authors:** Jordan Rempel, Chukwunonso Onyilagha, Kalhari Goonewardene, Aruna Ambagala

**Affiliations:** ^1^ National Centre for Foreign Animal Disease, Canadian Food Inspection Agency, Winnipeg, MB, Canada; ^2^ Department of Medical Microbiology and Infectious Diseases, Max Rady College of Medicine, University of Manitoba, Winnipeg, MB, Canada; ^3^ Comparative Biology and Experimental Medicine, Faculty of Veterinary Medicine, University of Calgary, Calgary, AB, Canada

**Keywords:** African swine fever, ASFV, inactivation, MTM, transport, nucleic acid stability, oral fluid

## Abstract

African swine fever (ASF) continues to devastate swine populations across the globe. The causative agent, ASF virus (ASFV), is very stable and can remain infectious over long periods of time especially in contaminated blood and tissue samples. Therefore, the transport of clinical samples from the field to diagnostic laboratories requires special precautions to reduce the risk of spreading the disease. Inactivation of ASFV in the clinical samples prior to transporting to the lab eliminates the risk and the requirement of higher biosafety facilities to perform ASF diagnostics. This study evaluated the use of a commercial molecular transport medium (MTM) to inactivate ASFV and stabilize the viral DNA in cell culture and clinical samples collected from pigs inoculated with different ASFV strains. In all the sample types tested, complete inactivation of ASFV was observed, without affecting the subsequent detection of ASFV genomic material by real time polymerase chain reaction (real time PCR). The MTM preserved the stability of ASFV genomic material eliminating the need to refrigerate clinical samples. The data shows that the MTM can be used reliably to ensure safety and stability of routine clinical samples such as whole blood, spleen swabs and alternative sample types such as oral fluid, allowing expansion and streamlining ASF molecular diagnostics.

## 1 Introduction

African swine fever virus (ASFV) is a large double-stranded DNA virus of the *Asfarviridae* family. ASFV causes African swine fever (ASF), a lethal hemorrhagic disease in domestic and wild Eurasian pigs (Dixon et al., 2019). ASFV is stable and remain infectious for a long period of time when protected in organic material and stored at lower temperatures (Mazur-Panasiuk et al., 2019). ASFV persists in blood for over a year at 4°C, and multiple weeks in pork products (Fischer et al., 2020). ASF emerged from ancient animal wildlife reservoirs in East Africa where it was first reported in Kenya in 1921, and since then it has been reported in over 80 countries across Africa, Americas, Asia, and Europe (Dixon et al., 2020; Li et al., 2022; WOAH, 2024). Transmission of ASFV can occur with direct contact between pigs by oral-nasal route, and indirectly via materials or feed contaminated with infected blood or other secretions, including feces, urine, and saliva (Penrith and Vosloo, 2009; Guinat et al., 2016; Dixon et al., 2019; Mazur-Panasiuk et al., 2019). Outbreaks of ASF can lead to considerable economic and domestic product losses in affected countries, as well as devastating impacts in rural regions that heavily rely on pork and pork products as a major part of their income and food security (You et al., 2021; Jean-Pierre et al., 2022). Currently, there is no cure for ASF. There have been promising strides towards an effective vaccine, however, no globally accepted vaccine is available for ASF. Therefore, implementation of basic biosecurity and early detection are crucial for combatting this dreadful disease in order to reduce the need for containment and elimination of infected and at-risk animals (Dixon et al., 2019; Mazur-Panasiuk et al., 2019; Dixon et al., 2020; Fernandez-Colorado et al., 2024).

ASF is characterized by fever, lethargy, loss of appetite and hemorrhages. It cannot be differentiated from other hemorrhagic diseases in pigs by either clinical signs or post-mortem examinations. Hence laboratory confirmation is critical. Real time polymerase chain reaction (PCR) is the most specific and sensitive assay currently available for rapid detection of ASFV genomic material in clinical samples, however it requires specific instruments, additional infrastructure, and highly skilled staff. Therefore, the samples from ASF suspected animals need to be delivered to a central laboratory for testing.

Whole blood is the ideal sample type from live pigs, and recent work has shown that spleen swabs rather than whole tissue homogenates could prove an effective sample type for ASFV detection in deceased pigs (Cafariello et al., 2024). The transport of clinical samples to the central laboratories presents a risk of spreading the infection and often requires maintaining cold chain during transportation to preserve the sample integrity. Maintaining cold chain adds extra costs and complications, especially for the countries with limited resources (Waldrup and Conger, 2002; Olsen, 2013). We recently showed that the spleen swabs are more sensitive than spleen tissue homogenates for ASFV genome detection (Cafariello et al., 2024). Spleen swabs can be easily collected and sent to diagnostic laboratories and processed quickly without the need for homogenization, and therefore suitable for high throughput detection. In another study, we have shown that swine oral fluid is a convenient and cost-effective sample type for the early detection of ASF (Goonewardene et al., 2021). Oral fluid collection is simple and straightforward and therefore can be performed by the farmers and submitted to diagnostic laboratory. However, unlike whole blood and spleen swabs, oral fluids are often contaminated with feces, soil, and feed particles, and therefore immediate refrigeration after collection is required to obtain reliable results.

PrimeStore^®^ molecular transport medium (MTM) is a commercially available reagent that contains ethanol, n-lauroylsarcosine, and guanidine thiocyanate, which lyse the cells and viruses while denaturing proteins such as nucleases, reducing degradation of nucleic acids (McGookin, 1985; Molina-Moya et al., 2020; Daum and Fischer, 2021; Welch et al., 2024). Following treatment with MTM, nucleic acid can be extracted by routine extraction methods including manual spin columns or automated magnetic bead extraction. MTM has been authorized by the Food and Drug Administration (FDA) for collection of samples suspected of containing Influenza A virus, and *Mycobacterium tuberculosis* (Daum and Fischer, 2021). Additional work has been done to demonstrate that MTM is able to inactivate Newcastle disease, eastern equine encephalitis, foot-and-mouth disease, bovine viral diarrhea, swinepox, vaccinia, Monkeypox, and SARS-CoV2 viruses (Molina-Moya et al., 2020; Daum and Fischer, 2021; Blacksell et al., 2023; Welch et al., 2024). To our knowledge, the ability of MTM to inactivate ASFV has not been reported. In this study, we evaluated the ability of MTM to inactivate ASFV and stabilize the ASFV nucleic acid in cell culture supernatant and clinical samples.

## 2 Materials and methods

### 2.1 Cells

For virus propagation and titration, highly ASFV-susceptible immortalized porcine kidney macrophage (IPKM) cell line was used (Masujin et al., 2021; Kameyama et al., 2022). IPKM cells develop clear cytopathic effect when infected with ASFV and the detection limit of the assay is almost identical to that of the porcine alveolar macrophage based classical hemadsorption assay. IPKM cells were maintained and passaged in Dulbecco’s modified Eagle medium (DMEM), high glucose, supplemented with 10% (v/v) non-irradiated fetal bovine serum (FBS), 2% (v/v) 200mM L-Glutamine, 1.1% (v/v) sodium pyruvate, 1% (v/v) Pen/Strep, 10µg/mL bovine insulin, 25µM monothioglycerol, and 0.022% (v/v) Fungicin (complete medium) in cell culture plates and flasks by the National Center for Foreign Animal Disease Reagent Development Unit (NCFAD-RDU). Cells were incubated at 37°C with 5% CO_2_ with complete medium for maintenance conditions. For infections, they were seeded 48 hours prior to use and infected at 90% confluency.

### 2.2 Samples

Cell culture supernatants containing ASFV strains belonging to three different p72 genotypes and archived whole blood, spleen, and oral fluid samples collected from pigs infected with different ASFV strains were used (**Table 1**). Cell culture supernatants were prepared by propagating the virus in primary pig leukocytes (PPLs) followed by titration on primary alveolar macrophages (PAMs) as previously described (Ambagala et al., 2024).

**Table 1:** Description of the types of samples and the viruses used in the study.

Sample type	Virus used	p72 genotype	Virulence	Reference
Culture sup.	ASFV Dom. Republic DR84	II	Moderate	(Schambow et al., 2025)
Culture sup.	ASFV Vietnam VNUA/rASFV/VP1/2023	I/II	High	(Le et al., 2024)
Culture sup.	ASFV Uganda/DA11121-36	IX	**	(Okwasiimire et al., 2023)
Culture sup.	ASFV Zambia/2019-SLNP-24	VIII	High	(Chambaro et al., 2020)
Blood/Culture sup.	ASFV Georgia 2007/1	II	High	(Rowlands et al., 2008)
Blood/Culture sup.	ASFV Nigeria RV502	II	High	(Ambagala et al., 2023)
Blood	ASFV Ghana Akuse	I	High	(Rai et al., 2024)
Culture sup.	ASFV Lillie	XX	High	(Boshoff et al., 2007)
Spleen swabs	ASFV Estonia 2014	II	Moderate	(Zani et al., 2018)
Oral fluid/Blood/Culture sup.	ASFV Malta’78	I	Moderate	(Wilkinson et al., 1980)
Culture sup.	ASFV OURT 88/3	I	Low	(Boinas et al., 2004)
Culture sup.	ASFV-G-ΔMGF-DMAC	II	Low	(Diep et al., 2025)

**Virulence unknown.

Culture sup: cell culture supernatant.

### 2.3 Molecular transport medium

PrimeStore^®^ MTM was purchased from Longhorn Vaccines and Diagnostics, LLC, Bethesda, MD, stored at room temperature and used as recommended (three parts of MTM to one part of sample, incubated for 60 minutes) unless otherwise noted.

### 2.4 Detergent removal and cytotoxicity testing

To reduce the cytotoxicity of MTM, Pierce^®^ detergent removal spin columns (Thermo Fisher Scientific, Waltham, MA, USA. Catalog number 87778) were used according to the manufacturer’s instructions. Briefly, the bottoms of the columns were snapped off, placed into sterile 15 mL falcon tubes, and centrifuged 1,000 x g for 2 minutes at room temperature to remove storage buffer. Columns were then washed 3 times with 2 mL of sterile Dulbecco’s phosphate buffered saline (D-PBS), and centrifuged at 1,000 x g for 2 minutes at room temperature. Columns were then placed in a new 15 mL falcon tube, and 500 µL of the samples treated with either D-PBS or MTM was added to the columns, allowed to sit for at least 1 minute, then centrifuged at 1,000 x g for 2 minutes at room temperature.

The removal of cytotoxicity was assessed using the CyQUANT™ MTT Cell Viability Assay (Thermofisher Scientific, Catalog number V13154). D-MEM high glucose medium supplemented with 1% (v/v) 5mg/mL gentamicin was used as the diluent. One part diluent was added to 3 parts of MTM, vortexed for 10 seconds, and half of the volume was subjected to detergent removal process, while the other half was used as is. The two treatments were serially diluted (from 10^−1^ to 10^-8^) in diluent. Cell culture medium was removed from 96-well IPKM plates, and 50 µL of dilutions were added to the plates, with 8 replicates per dilution. Plates were incubated at 37°C with 5% CO_2_ for 1 hour, and 200 µL of complete medium was added to each well. Plates were then incubated at 37°C with 5% CO_2_ for 48 hours, after which the medium was removed. Plates were washed 2 times with 200 µL warm D-PBS, and 100 µL of fresh complete medium, free of phenol-red, was added to each well. MTT substrate was then reconstituted in 1 mL of sterile D-PBS, and 10 µL was added to each well, and the plates were incubated at 37°C with 5% CO_2_ for 4 hours. After the incubation, 100µL of the SDS-HCL solution was added to each well, mixed by pipetting and plates were incubated at 37°C with 5% CO_2_ for another 4 hours. Each well was mixed again by pipetting, and absorbance was read at 570nm using SpectraMax Plus 384 Microplate Reader (Molecular Devices). The absorbance of the wells without cells were averaged and subtracted from all other wells as background. The absorbance of untreated control cells was averaged and used as 100% viable cells for the corresponding plate. Replicates for each treatment were then averaged, and cell viability was calculated as shown below.


$$Cell\;viability\%=\frac{\left(OD_{570}Treated\;cells-OD_{570}Empty\;wells\right)}{\left(OD_{570}Control\;cells-OD_{570}Empty\;wells\right)}\text{x}100$$

### 2.5 Virus Titration

To evaluate virus titer reduction after the MTM treatment, IPKM cells grown to 90% confluency in 96-well tissue culture plates were used. The samples containing ASFV treated with either D-PBS or MTM were passed through the detergent removal spin columns as described above, and the eluate was serially diluted (10-fold) in DMEM high glucose medium supplemented with 1% (v/v) 5mg/mL gentamicin up to 10^-8^. Just before virus inoculation, cell culture medium was removed from the 96-well plates, and each well was inoculated with 50 µL of serially diluted samples. Plates were then incubated at 37°C with 5% CO_2_ for 1 hour, after which 150µL of complete cell culture medium was added to each well, and plates were incubated at 37°C with 5% CO_2_. Plates were observed daily up to 7 days for cytopathic effect (CPE), after which virus TCID_50_ was calculated. Titrations were performed in duplicate. Virus titer reduction in MTM treated samples was compared to the controls treated with D-PBS.

### 2.6 Virus Isolation

For virus isolation, IPKM cells grown to 90% confluency in T25 flasks were used. The cell culture medium was removed from the T25 flasks, and 0.5 mL of the above prepared 10-fold diluted eluate was added to each flask. The flasks were then incubated at 37°C with 5% CO_2_ for 1 hour with gentle rocking every 15 minutes; after the 1-hour incubation, the flasks were topped off with 4.5 mL complete medium. The flasks were incubated at 37°C with 5% CO_2_ for up to 7 days and observed daily for CPE. On the 7^th^ day, the flasks were frozen at -70°C for minimum of 4 hours before being placed in 37°C incubator to thaw. The contents of the flask were transferred into a 15 mL falcon tube, centrifuged at 2,000 x g for 20 minutes at 4°C, and the supernatant was used for nucleic acid extraction for ASFV real time PCR, and for the next passage following the same procedure, up to 3 passages.

### 2.7 Evaluation of MTM on the stability of ASFV nucleic acid

Cell culture supernatant, pig blood, and oral fluids containing different ASFV strains (**Table 1**) were treated with MTM or D-PBS. Spleen swabs were collected in duplicates with sterile polyester-tipped applicator swabs (Puritan #25-806- 1PD, Puritan Medical Products, Falmouth, ME, USA) as described previously (Cafariello et al., 2024). The collected swabs were placed into 2 mL clear cryovials containing 1.25 mL of sterile D-PBS, and the excess lengths of the swabs were snapped off. The cryovials were then closed and vortexed for 10 seconds; the duplicates were pooled, vortexed briefly, and then aliquoted for treatment with either MTM or D-PBS. Treated samples were then aliquoted into separate tubes for incubation at either room temperature or 4°C, and nucleic acid was extracted at 0, 3-, 7-, 14-, and 21-days following incubations.

### 2.8 Nucleic acid extraction and real time PCR for detection of ASFV genomic material

ASFV genomic material was extracted using MagMax™ Pathogen DNA/RNA kit (Thermofisher Scientific Cat. No. 4462359) and Kingfisher Apex purification system (Thermo Fisher). For nucleic acid extraction, 55 µL of blood or cell culture amplified virus was used; 200 µL of spleen swabs or oral fluids was used as per the NCFAD low cell count nucleic acid extraction protocol. Tignon ASFV real time PCR assay, which targets a highly conserved region of the ASFV p72 open reading frame was used for ASFV genome detection and quantification (Tignon et al., 2011). Beta-actin real time PCR was used as the internal control for the samples and all assays were run on Bio-Rad CFX96 Touch Real-Time PCR Detection System (Bio-Rad, Mississauga, ON, Canada), using recommended cycling conditions for TaqMan™ Fast Virus 1-Step Master Mix, up to 40 cycles. Cycle threshold (Ct) values were determined by regression analysis (CFX Maestro Software for CFX Real-Time PCR Instruments, Bio-Rad) of a positive control plasmid, and the cutoff threshold was assigned based on this value for all samples in the plate. Samples with Ct values 35.44 and below were considered positive; samples with Ct values ranging from 35.45 to 39.99 were considered suspicious, while samples with Ct values of 40 and above were considered negative.

## 3 Results and discussion

### 3.1 MTM-induced IPKM cytotoxicity can be reduced by detergent removal spin columns

**Figure 1:**
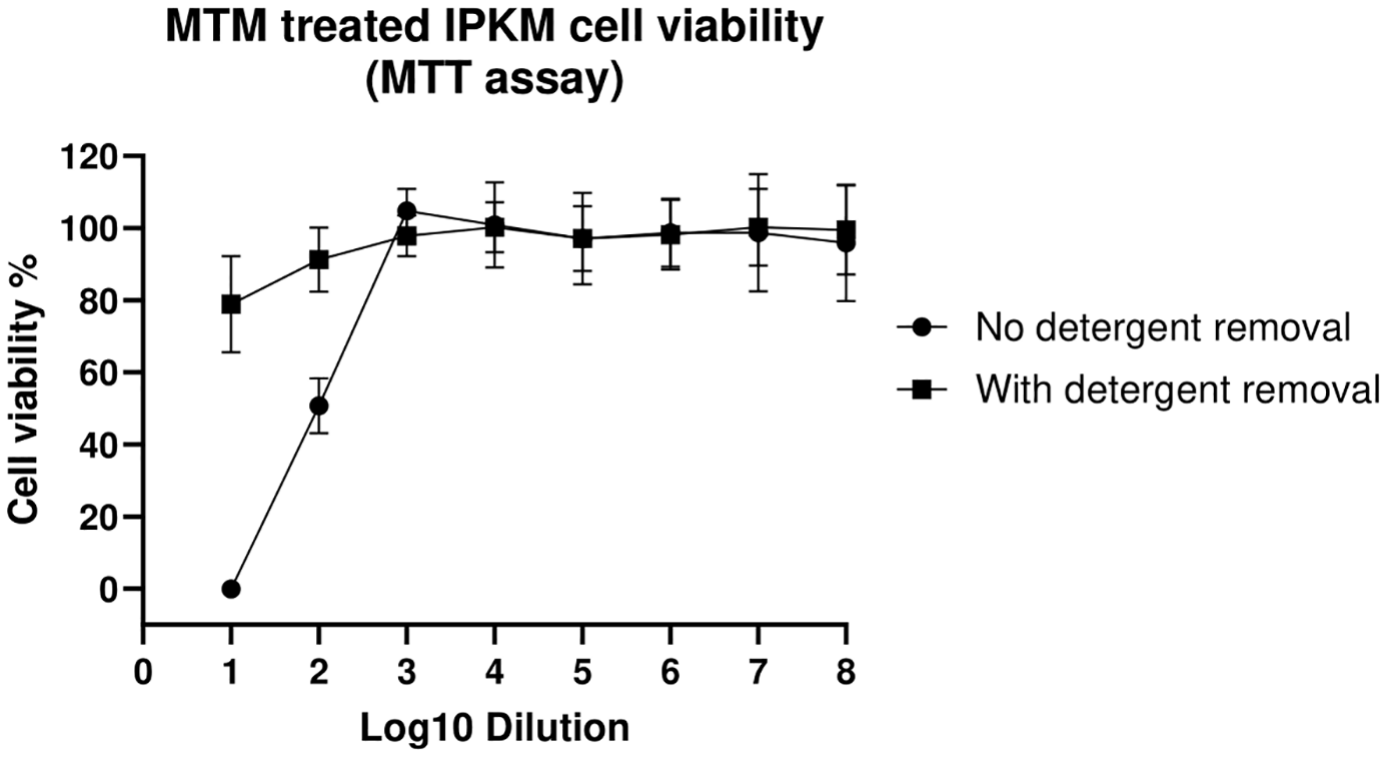
The detergent spin columns reduce cytotoxicity associated with MTM. Cell viability was assessed using the MTT assay, where cells were treated (48 hours) with varying concentrations of MTM with or without the use of detergent removal columns. Absorbance was measured at 570nm, and replicates were averaged to determine cell viability. The figure shows the percentage of viable cells relative to untreated cell controls. Error bars represent the standard deviation between replicates.

Viability of IPKM cells after the addition of MTM treated samples was evaluated using the CyQUANT™ MTT Cell Viability Assay and visually under a light microscope. When the IPKM cells were exposed to 10-fold diluted MTM treated sample, all the cells were killed (0% viability) by 48 hours (**Figure 1**). In contrast, IPKM cells exposed to 10-fold diluted, detergent purified MTM treated sample, showed ~80% viability. When samples treated with MTM were diluted 100-fold, and no detergent removal columns were used, IPKM cells showed 50% viability, but at 1,000-fold dilutions and higher, MTM was not toxic to the IPKM cells, and the cells appeared healthy and remained as an intact monolayer until the end of the experiment at 48 hours.

### 3.2 MTM at the recommended ratio, completely inactivates ASFV within 10 minutes

**Figure 2:**
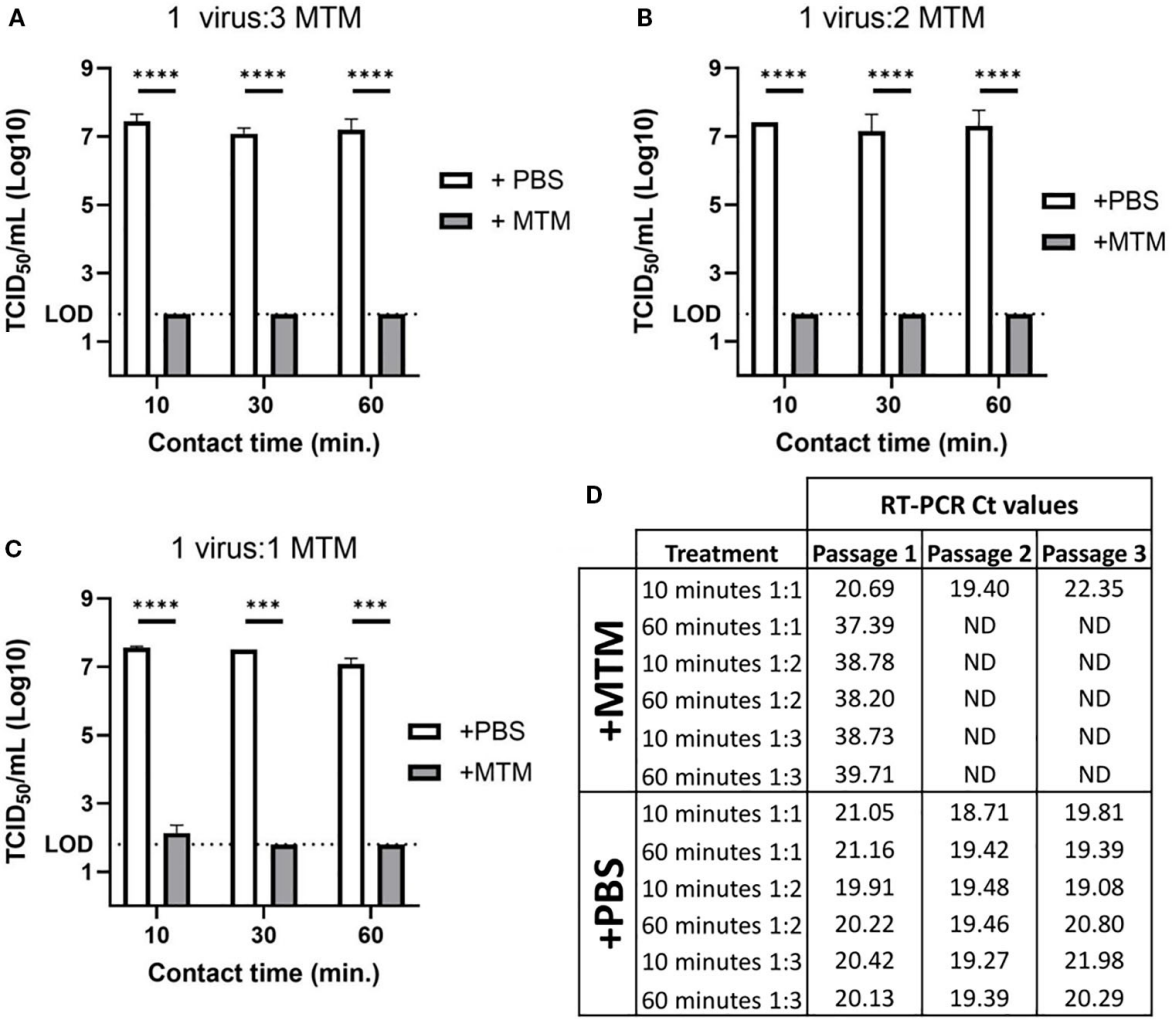
ASFV titer reduction in cell culture supernatant following MTM treatment. Cell culture amplified ASFV Georgia 2007/1 was treated with molecular transport medium (+MTM) or PBS control (+PBS) at varying ratios for 10, 30, or 60 minutes. **(A)** MTM reduced ASFV titer below the limit of detection in as little as 10 minutes at a ratio of 1-part virus to 3-parts MTM. **(B)** The same was seen with 1 part virus to 2 parts MTM at 10, 30, and 60 minutes. **(C)** At 1 part virus to 1-part MTM, ASFV titer was below the limit of detection after 30 and 60 minutes, but after 10 minutes there was still live virus that was seen with viral titration. **(D)** Complete viral inactivation was confirmed by viral isolation over three passages, and real time PCR data showed no detectable viral genomic material at second passage with all MTM treated samples, except for 10 minutes with 1-part virus to 1-part MTM, which had similar levels of genomic material detected at each passage, indicating consistent viral growth. Significance was determined using Student’s t-test ***p< 0.001; ****p< 0.0001; ND, not detected.

To demonstrate that MTM at the recommended concentration (1 part sample to 3 parts of MTM, and a minimum incubation period of 60 minutes) can completely inactivate ASFV, cell culture amplified highly virulent ASFV Georgia 2007/1 was used. MTM treatment reduced the titers of ASFV Georgia 2007/1 below the limit of detection (LOD, 10^1.80^ TCID_50_ calculated based on improved Kärber method) in virus titration assay, (**Figure 2A**).

Under field conditions, maintaining the exact sample to MTM ratio (1:3) could be challenging during sampling; therefore, we evaluated the ability of MTM to inactivate ASFV at different sample to MTM ratios (1:2 and 1:1). To determine the minimum time to inactivate ASFV at those ratios, the samples were incubated for 10-, 30-, and 60-minutes. Sample treatment with 1:3 and 1:2 sample to MTM ratios reduced virus titers to below the limit of detection in the virus titration assay within 10 min (**Figures 2A, B**). When MTM was added to sample at a 1:1 ratio, more than 5-log titer reduction was observed; however, live virus was present at very low levels after the 10 minutes treatment (**Figure 2C**). ASFV was isolated from those samples after the first passage on T25 flasks and the virus continued to grow in subsequent passages, indicating the inability of MTM to completely inactivate ASFV at 1:1 under 10 minutes, while conversely, no virus was isolated in samples treated with increased MTM ratios, or prolonged exposure time with MTM in any subsequent passages (**Figure 2D**). All samples treated with D-PBS had stable Ct values, and showed CPE at every passage, indicating continual viral growth in these samples.

Each treatment was done in duplicate and therefore virus isolation was carried out only on the samples incubated for 10 and 60 minutes to manage the resources.

### 3.3 MTM inactivation of ASFV is not strain- or sample type-specific

**Figure 3:**
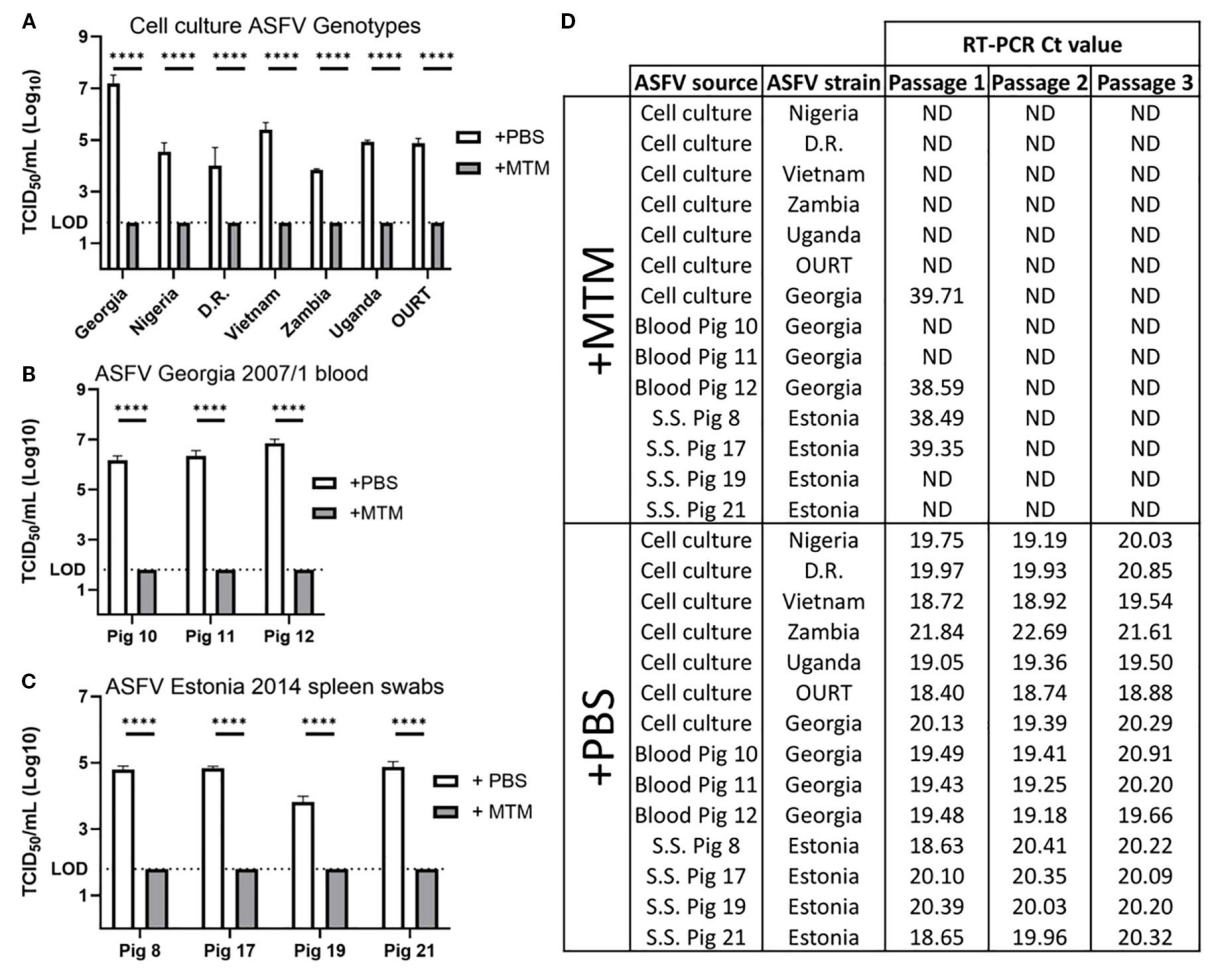
Inactivation of different ASFV genotypes in cell culture and clinical samples. ASFV samples were treated with MTM (+MTM) or PBS control (+PBS) at 3:1 ratio for 60 minutes. **(A)** MTM inactivated ASFV below the limit of detection, up to 5-log titer reduction, regardless of genotype. **(B)** Whole blood samples from pigs experimentally infected with ASFV Georgia 2007/1 were treated with MTM, and in all cases, MTM reduced ASFV titers below the limit of detection. **(C)** Spleen swabs from 4 different pigs experimentally infected with ASFV Estonia 2014 were treated with MTM, and in all cases, MTM reduced ASFV titers below the limit of detection **(D)** To Ensure complete viral inactivation, MTM treated samples were passaged 3 times on IPKM cells, and at second and third passage, no genomic material could be detected in any samples treated with MTM, indicating complete viral inactivation. Statistical significance was determined using Student’s t-test ****p< 0.0001; ND, not detected; D.R, Dominican Republic; S.S., spleen swabs.

To determine if MTM completely inactivates ASFV strains other than ASFV Georgia 2007/1, 6 additional cell culture amplified ASFV strains were treated with MTM for 60 min. MTM treatment reduced the virus titers to undetectable levels (**Figure 3A**), indicating that MTM can inactivate not only the historic p72 genotype II ASFV Georgia 2007/1 strain, but also more recent ASFV genotype II strains such as Vietnam VNUA/rASFV/VP1/2023, Nigeria RV502, Dominican Republic DR84, and genotype IX ASFV Uganda/DA11121-36, and genotype VIII ASFV Zambia/2019-SLNP-24.

Whole blood (EDTA) is the best clinical sample for early detection of ASF. Pigs infected with highly virulent strains develop viremia and the virus titers in the blood can reach up to 10^9^ HAD_50_/mL (Jaing et al., 2017). Such a high virus titer makes it the ideal sample for ASFV genome detection but also considered extremely risky during transportation from the field to central laboratories. The ability to inactivate live virus in blood eliminates the risk of transporting samples with high titered virus. To determine if MTM can inactivate ASFV in whole blood, samples collected at the peak of viremia from three different pigs infected with highly virulent ASFV Georgia 2007/1 were treated with MTM (1-part virus to 3-part MTM) or D-PBS and subjected to virus titration and isolation. All three blood samples, when treated with MTM, showed reduced viral titers below the LOD (>4-log titer reduction) compared to the D-PBS treated controls (**Figure 3B**).

In addition to the whole blood, the ability of MTM to inactivate ASFV in spleen swabs was also evaluated using the spleen swabs collected from four pigs that succumbed to the naturally attenuated ASFV Estonia 2014 (genotype II). Spleen swabs treated with MTM showed a titer reduction ranging from >2-log to >3-log (**Figure 3C**). All sample types were also passaged 3 times in IPKM cells. In all cases, no CPE was observed or genomic material detected in the cell culture amplified virus, whole blood, and spleen swab samples treated with MTM by the third passage, unlike the D-PBS treated control samples that had consistent CPE and decreasing or stable Ct values. (**Figure 3D**).

### 3.4 MTM stabilizes ASFV nucleic acid in clinical samples

**Figure 4:**
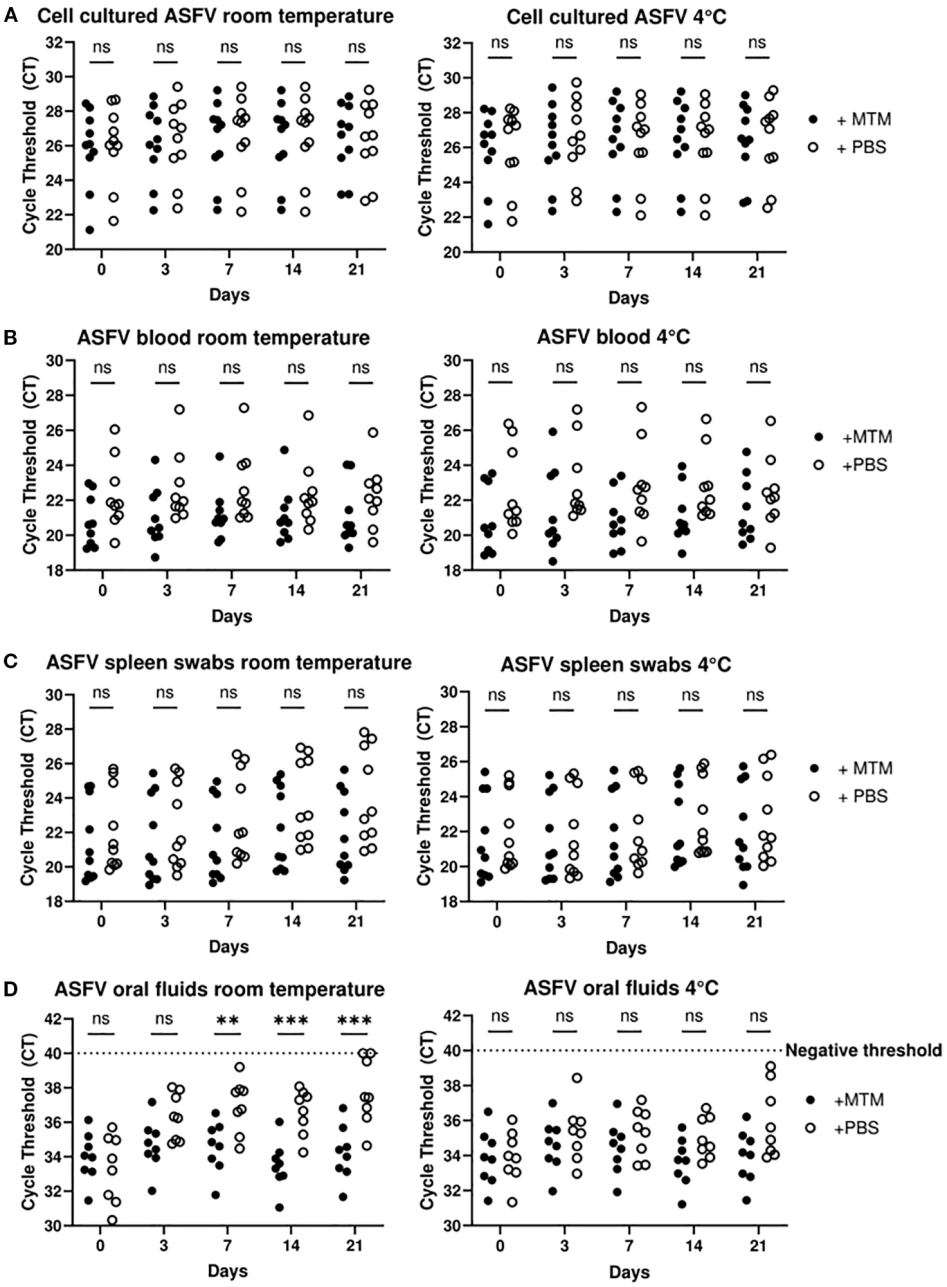
MTM stabilizes ASFV genomic material in multiple sample types. Cell culture supernatant containing ASFV Lillie, OURT 88, Malta 78’, Georgia 2007/1, Nigeria RV502, ASFV-G-ΔMGF-DMAC or Dom. Republic DR84 **(A)**, whole blood containing ASFV Nigeria RV502 or Ghana Akuse **(B)**, spleen swabs containing ASFV Estonia 2014 **(C)** and oral fluid containing ASFV Malta 78’ **(D)** were treated with MTM (+MTM) or D-PBS (+PBS). Nucleic acid was extracted and subjected to ASFV real time PCR and the Ct values of MTM treated samples were compared to that treated with PBS. The data show MTM stabilizes ASFV genomic material in clinical samples specially in oral fluids at room temperature. Significance was determined using Student’s t-test **p< 0.01; ***p< 0.001, ns, not significant.

The ability to transport inactivated ASFV samples to central laboratories at room temperature with no deleterious effect on ASFV nucleic acid is critical for reliable results. We assessed the ability of MTM to stabilize ASFV genomic DNA at room temperature and 4°C. In this study, 10 cell culture amplified virus samples, 10 porcine spleen swab samples, 9 blood samples, and 8 oral fluid samples containing ASFV were tested at 0, 3, 7, 14 and 21 days after treatment. Nucleic acid was extracted at indicated time points and subjected to ASFV real time PCR. The real time PCR results from cell culture amplified virus were stable at all time points, with no significant change of Ct values during the time course. There was also no change when samples were stored at room temperature or 4°C, with or without MTM (**Figure 4A**). The same pattern was observed with blood, with no significant change in Ct values between the samples treated with MTM or D-PBS (**Figure 4B**). Interestingly, infected blood samples treated with MTM consistently showed lower Ct values at every time point compared to their D-PBS treated counterparts; although the exact reason for this is unclear, we speculate that this could be a result of MTM lysing the infected cells in the blood prior to the lysing step during extraction, which may increase nucleic acid extraction yield.

Spleen swabs treated with MTM did show adequate stability at both room temperature and 4°C, however swabs treated with D-PBS were not as stable, showing up to 2 Ct values increase after 21 days at room temperature compared to the MTM treated counterparts (**Figure 4C**). Though this was not a statistically significant difference, there was a gradual loss of detectable ASFV genomic material over the time course; should we have started with a sample with a high Ct value, this could have resulted in a loss of detectable genomic material after a prolonged period. When the swabs were stored at 4°C in D-PBS, the average Ct value change was less than 1 over the time course, indicating that there was no loss of ASFV genome integrity for real time PCR detection.

Oral fluids indicated significantly improved stability of samples when treated with MTM at room temperature. After 21 days at room temperature, the D-PBS samples saw an average increase of over 3 Ct values, with some values beyond the negative threshold and not detectable by real time PCR. When looking at oral fluids at 4°C, samples treated with D-PBS were more stable compared to room temperature, but on average there was still a decrease of nearly 2 Ct values at the end of the time course (**Figure 4D**). All samples treated with MTM varied by less than 1 Ct value on average at room temperature and 4°C. For cell amplified virus, infected blood, and spleen swabs, there was no significant difference when treated with D-PBS in both temperature conditions. The most significant increase in stability was seen with oral fluids at room temperature when treated with MTM compared to D-PBS. This could have been partly due to the unclean complex nature of oral fluid matrix compared to other simpler sample matrices used here. Swine oral fluids contain a spread of microbial diversity, some of which may produce extracellular deoxyribonucleases, thereby degrading DNA and reducing detectable levels of genomic material (Sumby et al., 2005; Hattab et al., 2021; Buiatte et al., 2024). Since MTM denatures proteins such as DNAses, and inactivates a range of infectious pathogens, it is likely inactivating most of the microbial contaminants found in the swine oral fluids, thereby inhibiting the degradation of the genomic material and increasing overall stability.

## 4 Conclusions

In this study, we have shown that a commercially available molecular transport medium, PrimeStore™ MTM, at the recommended concentration, completely inactivates ASFV in different clinical materials and preserves the nucleic acid for subsequent detection by real time PCR. We also showed that MTM effectively inactivates ASFV, regardless of the genotype, and maintains the nucleic acid stability at ambient temperatures, especially with samples heavily contaminated with bacteria, such as oral fluids. Whole blood and oral fluid can be added to MTM at 1:3 ratio and spleen swabs can be collected in a tube containing PBS and diluted in MTM at 1:3 ratio. When using spleen swabs, there is increased assay sensitivity compared to spleen homogenates (Cafariello et al., 2024); therefore, spleen swabs can be collected during postmortems in the field, added to MTM, and shipped to the central laboratories for ASFV genome detection. The swabs can be processed immediately upon arrival for quick result turnaround time.

Complete inactivation of ASFV in clinical samples eliminates the risk of transporting clinical samples containing live ASFV across ASF-free areas, and the ability to ship the samples at ambient temperature eliminates the need to maintain cold chain during transportation and storage. The ability to transport and store clinical samples at ambient temperatures until laboratory testing is finalized reduces the financial constraints that come with cold chain storage. Since MTM completely inactivates ASFV, the samples can also be processed at BSL2 laboratories allowing decentralization of front-line molecular testing (real time PCR) during an ASF outbreak, decreasing the reporting time and increasing the overall laboratory testing capacity.

In addition to clinical samples, MTM can also be used to prepare safe and stable interlaboratory proficiency panels for molecular detection of ASF. MTM treatment will allow transport of proficiency panels at room temperature, thereby significantly reducing the shipping cost, which is one of the biggest challenges faced by countries with limited resources.

The main limitation of MTM treatment of clinicals samples is that the treated sample can’t be used for virus isolation. At the beginning of an ASF incursion, virus isolation is critical for the determination of hemadsorption (HAD) phenotype and the pathotype of the virus. Therefore, MTM treatment is not ideal to use on the samples (for virus isolation) collected during surveillance or initial suspicion of an ASF incursion. However, once an ASF outbreak is declared, the use of MTM to treat the clinical samples collected at affected farms would allow safe, reliable, and low-cost transport of clinical samples from the field, further decentralizing, expanding, and streamlining ASF molecular diagnostics.

In this study solid tissues (lymph nodes, spleen etc.) were not evaluated since the penetration of MTM into the tissues may vary depending on the type, density and size of the tissues, however it warrants further investigation.

## Data Availability

The raw data supporting the conclusions of this article will be made available by the authors upon request, without undue reservation.
